# Supplementary material for: Multi‐dataset identification of innovative feature genes and molecular mechanisms in keratoconus
Source: J Cell Mol Med. 2024 Sep 19;28(18):e70079. doi: 10.1111/jcmm.70079 (PMC11412914; doi:10.1111/jcmm.70079)
Supplement: Supplementary file 2 — Material S2. [file JCMM-28-e70079-s002.zip › 7.GSEA analysis/5.RNF217-AS1/KEGG_COLORECTAL_CANCER.html]

Details for gene set KEGG\_COLORECTAL\_CANCER[GSEA]

|  || Dataset | RNF217-AS1.group.cls#h\_versus\_l |
| Phenotype | group.cls#h\_versus\_l |
| Upregulated in class | l |
| GeneSet | KEGG\_COLORECTAL\_CANCER |
| Enrichment Score (ES) | -0.43767577 |
| Normalized Enrichment Score (NES) | -1.7433215 |
| Nominal p-value | 0.011952192 |
| FDR q-value | 0.56666374 |
| FWER p-Value | 0.292 |
Table: GSEA Results Summary

  

Fig 1: Enrichment plot: KEGG\_COLORECTAL\_CANCER      
 Profile of the Running ES Score & Positions of GeneSet Members on the Rank Ordered List

  

| SYMBOL | TITLE | RANK IN GENE LIST | RANK METRIC SCORE | RUNNING ES | CORE ENRICHMENT || 1 | PIK3R5 | na | 1058 | 0.434 | 0.0296 | No |
| 2 | BRAF | na | 5517 | 0.199 | -0.0715 | No |
| 3 | RALGDS | na | 6708 | 0.162 | -0.0835 | No |
| 4 | PIK3CD | na | 6778 | 0.159 | -0.0634 | No |
| 5 | RAC3 | na | 7163 | 0.148 | -0.0540 | No |
| 6 | TGFB1 | na | 9166 | 0.102 | -0.0977 | No |
| 7 | CASP3 | na | 9912 | 0.088 | -0.1070 | No |
| 8 | GSK3B | na | 10962 | 0.071 | -0.1275 | No |
| 9 | PIK3CB | na | 11439 | 0.064 | -0.1324 | No |
| 10 | KRAS | na | 11445 | 0.064 | -0.1238 | No |
| 11 | MSH3 | na | 11551 | 0.062 | -0.1182 | No |
| 12 | MAPK9 | na | 12895 | 0.044 | -0.1509 | No |
| 13 | TCF7L2 | na | 12940 | 0.043 | -0.1463 | No |
| 14 | RAC2 | na | 12945 | 0.043 | -0.1405 | No |
| 15 | MAPK1 | na | 13362 | 0.038 | -0.1472 | No |
| 16 | PIK3CA | na | 14021 | 0.031 | -0.1620 | No |
| 17 | MAPK3 | na | 14127 | 0.029 | -0.1610 | No |
| 18 | SMAD3 | na | 14794 | 0.021 | -0.1772 | No |
| 19 | RHOA | na | 14935 | 0.020 | -0.1785 | No |
| 20 | DCC | na | 15126 | 0.018 | -0.1816 | No |
| 21 | AXIN1 | na | 15329 | 0.016 | -0.1852 | No |
| 22 | MAPK10 | na | 15620 | 0.013 | -0.1918 | No |
| 23 | AKT2 | na | 15816 | 0.011 | -0.1960 | No |
| 24 | SMAD2 | na | 16024 | 0.008 | -0.2008 | No |
| 25 | PIK3R1 | na | 16145 | 0.007 | -0.2032 | No |
| 26 | APPL1 | na | 16161 | 0.007 | -0.2027 | No |
| 27 | BAD | na | 16311 | 0.006 | -0.2062 | No |
| 28 | TCF7L1 | na | 16424 | 0.004 | -0.2088 | No |
| 29 | MAPK8 | na | 16681 | 0.002 | -0.2159 | No |
| 30 | SMAD4 | na | 16849 | 0.000 | -0.2207 | No |
| 31 | AKT1 | na | 18280 | -0.013 | -0.2601 | No |
| 32 | RAC1 | na | 18357 | -0.014 | -0.2603 | No |
| 33 | TGFBR2 | na | 18634 | -0.017 | -0.2660 | No |
| 34 | CCND1 | na | 18820 | -0.019 | -0.2688 | No |
| 35 | RAF1 | na | 18969 | -0.020 | -0.2703 | No |
| 36 | CTNNB1 | na | 19818 | -0.028 | -0.2909 | No |
| 37 | TCF7 | na | 19939 | -0.029 | -0.2903 | No |
| 38 | CASP9 | na | 20912 | -0.039 | -0.3129 | No |
| 39 | BAX | na | 22034 | -0.051 | -0.3382 | No |
| 40 | MSH2 | na | 22289 | -0.054 | -0.3381 | No |
| 41 | CYCS | na | 22416 | -0.055 | -0.3341 | No |
| 42 | PIK3R2 | na | 22894 | -0.061 | -0.3395 | No |
| 43 | MAP2K1 | na | 23215 | -0.064 | -0.3398 | No |
| 44 | MSH6 | na | 23674 | -0.071 | -0.3433 | No |
| 45 | BIRC5 | na | 23833 | -0.073 | -0.3378 | No |
| 46 | MLH1 | na | 24245 | -0.079 | -0.3387 | No |
| 47 | TGFB2 | na | 24475 | -0.082 | -0.3340 | No |
| 48 | ARAF | na | 25299 | -0.095 | -0.3445 | No |
| 49 | FOS | na | 25888 | -0.105 | -0.3469 | No |
| 50 | JUN | na | 27259 | -0.131 | -0.3683 | No |
| 51 | TGFBR1 | na | 27639 | -0.140 | -0.3599 | No |
| 52 | PIK3CG | na | 30335 | -0.218 | -0.4076 | Yes |
| 53 | MYC | na | 30784 | -0.234 | -0.3882 | Yes |
| 54 | APC | na | 31337 | -0.256 | -0.3687 | Yes |
| 55 | AKT3 | na | 31832 | -0.281 | -0.3440 | Yes |
| 56 | TGFB3 | na | 32072 | -0.296 | -0.3100 | Yes |
| 57 | TP53 | na | 32227 | -0.304 | -0.2724 | Yes |
| 58 | PIK3R3 | na | 32895 | -0.352 | -0.2429 | Yes |
| 59 | BCL2 | na | 33060 | -0.365 | -0.1971 | Yes |
| 60 | LEF1 | na | 34024 | -0.487 | -0.1576 | Yes |
| 61 | APC2 | na | 34163 | -0.517 | -0.0901 | Yes |
| 62 | AXIN2 | na | 34629 | -0.769 | 0.0029 | Yes |
Table: GSEA details [plain text format]

  

Fig 2: KEGG\_COLORECTAL\_CANCER      
 Blue-Pink O' Gram in the Space of the Analyzed GeneSet

  

Fig 3: KEGG\_COLORECTAL\_CANCER: Random ES distribution      
 Gene set null distribution of ES for **KEGG\_COLORECTAL\_CANCER**
